# Association of sex and cardiovascular risk factors with atherosclerosis distribution pattern in lower extremity peripheral artery disease

**DOI:** 10.3389/fcvm.2023.1004003

**Published:** 2023-06-27

**Authors:** Oliver Baretella, Laura Buser, Claudine Andres, Dario Häberli, Armando Lenz, Yvonne Döring, Iris Baumgartner, Marc Schindewolf

**Affiliations:** ^1^ Division of Angiology, Swiss Cardiovascular Center, Inselspital, Bern University Hospital, University of Bern, Bern, Switzerland; ^2^ Clinical Trials Unit Bern, University of Bern, Bern, Switzerland; ^3^ Institute for Cardiovascular Prevention (IPEK), Ludwig-Maximilians-Universität (LMU) München, Munich, Germany; ^4^ German Centre for Cardiovascular Research (DZHK), Partner site Munich Heart Alliance, Munich, Germany; ^5^ Department for BioMedical Research (DBMR), Bern University Hospital, University of Bern, Bern, Switzerland

**Keywords:** lower extremity peripheral artery disease, iliac arteries, infragenicular arteries, diabetes mellitus, chronic kidney disease, smoking

## Abstract

**Objective:**

Atherosclerosis expression varies across not only coronary, cerebrovascular, and peripheral arteries but also within the peripheral vascular tree. The underlying pathomechanisms of distinct atherosclerosis phenotypes in lower extremity peripheral artery disease (PAD) is poorly understood. We investigated the association of cardiovascular risk factors (CVRFs) and atherosclerosis distribution in a targeted approach analyzing symptomatic patients with isolated anatomic phenotypes of PAD.

**Methods:**

In a cross-sectional analysis of consecutive patients undergoing first-time endovascular recanalization for symptomatic PAD, data of patients with isolated anatomic phenotypes of either proximal (iliac) or distal (infrageniculate) atherosclerosis segregation were extracted. We performed a multivariable logistic regression model with backward elimination to investigate the association of proximal and distal PAD with CVRFs.

**Results:**

Of the 637 patients (29% females) with endovascular recanalization, 351 (55%) had proximal and 286 (45%) had distal atherosclerosis. Female sex [odds ratio (OR) 0.33, 95% confidence interval (CI) 0.20–0.54, *p* = 0.01], active smoking (OR 0.16, 95% CI 0.09–0.28, *p* < 0.001), and former smoking (OR 0.33, 95% CI 0.20–0.57, *p* < 0.001) were associated with proximal disease. Diabetes mellitus (DM) (OR 3.25, 95% CI 1.93–5.46, *p* < 0.001), chronic kidney disease (CKD) (OR 1.18, 95% CI 1.08–1.28, *p* < 0.001), and older age (OR 1.31, 95% CI 1.06–1.61, *p* = 0.01) were associated with distal disease.

**Conclusion:**

Female sex, particularly in the context of smoking, is associated with clinically relevant, proximal atherosclerosis expression. Our additional findings that distal atherosclerosis expression is associated with DM, CKD, and older age suggest that PAD has at least two distinct atherosclerotic phenotypes with sex-specific and individual susceptibility to atherogenic risk factors.

## Introduction

Atherosclerosis is a chronic inflammatory disease of the arterial wall initiated by subendothelial plasma lipoprotein retention (1). Clinically, coronary artery disease (CAD), large vessel cerebrovascular disease (CVD), and lower extremity peripheral artery disease (PAD) can be distinguished depending on the affected vascular bed (2). Among these, PAD carries the highest known risk for cardiovascular complications (3, 4). While cardiovascular risk factors (CVRFs) impinge on the entire vascular bed, numerous studies have demonstrated that the distribution pattern of clinically relevant atherosclerosis across different vascular territories widely differs among patients and is distinctively associated with local site-specific as well as systemic cardiovascular risk factors (5–9). Hypercholesterolemia and an increased low-density lipoprotein cholesterol (LDL-C) emerge as strong risk factors for CAD, elevated systolic blood pressure for PAD and ischemic CVD, and smoking and diabetes mellitus (DM) for PAD (5, 7, 10, 11). Although PAD encompasses a large vascular territory with differences in diameter, vessel wall components, or hemodynamic characteristics, it is considered a single disease entity whether aortoiliac, femoropopliteal, or infrageniculate arteries are affected (12). Clinical observations suggest that there is also a distinct atherosclerosis pattern at predilection sites within the lower extremity vascular tree dependent on cardiovascular risk factor profiles (13, 14).

In the present study, we aim to describe the association of cardiovascular risk factors, clinical parameters, and atherosclerosis distribution patterns in a targeted approach analyzing patients with isolated anatomic phenotypes of peripheral atherosclerosis expression, i.e., proximal (iliac arteries) vs. distal (infrageniculate arteries) PAD undergoing first-time endovascular treatment.

## Materials and methods

### Patients

All patients of the Division of Vascular Medicine at Bern University Hospital, Division of Vascular Medicine, Swiss Cardiovascular Center, undergoing endovascular revascularization between January 2000 and March 2018 were assessed for study inclusion. We included all patients with clinically driven first-time endovascular recanalization of either proximal (iliac) or distal (infrageniculate) lower extremity arteries for chronic PAD (Rutherford stages 2–6) (12). Patients with multilevel disease, i.e., iliac, femoropopliteal, and/or infrageniculate requiring endovascular treatment of more than one segment were excluded from the study. Further exclusion criteria were intervention for acute limb ischemia and denial of general informed consent. Our database consists of clinically well-characterized PAD patients undergoing endovascular recanalization in a tertiary vascular center. Clinical and imaging data are routinely being entered by the treating physicians as part of the clinical treatment documentation in electronic data entry forms in a dedicated database [Clinic WinData (CWD), E&L Medical Systems GmbH, Erlangen, Germany]). Laboratory data and other health-related data are obtained from the laboratory system (ixserv, ix.mid Software Technologie GmbH, Cologne, Germany) and from the patients’ electronic medical records (i-pdos, CompuGroup Medical Schweiz AG, Bern, Switzerland). Other factors being documented are age, sex (based on biological features and medical health records), smoking status (active, former, never), arterial hypertension, body mass index (BMI), dyslipidemia, diabetes mellitus, chronic kidney disease (CKD), and C-reactive protein (CRP) as a surrogate marker of inflammation.

### Clinical assessment

Smoking was defined as tobacco use ≥1 pack year(s), for both active and former cigarette smoking, based on patient interview or chart documentation. Arterial hypertension was defined as systolic blood pressure >140 mmHg and/or diastolic blood pressure >90 mmHg and assumed if the patient was on antihypertensive therapy. Overweight and obesity were defined as a BMI of 25 to <30 and ≥30 kg/m^2^, respectively. Dyslipidemia was defined as total cholesterol level >5 mmol/L (193.4 mg/dl), or high-density lipoprotein cholesterol (HDL-C) level <1 mmol/L (38.7 mg/dl), triglyceride level >2 mmol/L (175 mg/dl), or LDL-C level >3.35 mmol/L (129.5 mg/dl), or assumed if the patient was on lipid lowering drugs (15, 16). Diabetes mellitus was defined as glycated hemoglobin (HbA_1c_) > 6.5%, fasting plasma glucose ≥7 mmol/L (126 mg/dl), post-challenge plasma glucose ≥11.1 mmol/L (200 mg/dl), or assumed if the patient was on glucose-lowering drugs (17). Chronic kidney disease was defined as an estimated glomerular filtration rate (eGFR) < 60 ml/min/1.73 m^2^, corresponding to chronic kidney disease stages ≥3. Laboratory values were considered if they were within a time window of 30 days of the primary intervention. Values with more than 30% missing were excluded from the analyses. Otherwise, variables were imputed assuming values to be missing at random. All variables were used to impute missing values in all other variables. In total, 20 imputed data sets were created, using predictive mean matching for continuous variables, logistic regression for binary variables, and the Bayesian polytomous regression model for categorical variables with more than two levels (i.e., smoking status). Due to the strong correlation among cholesterol variables, only HDL-C- and LDL-C values were included in the analysis, but not total cholesterol. LDL-C was calculated according to the Friedewald equation from total cholesterol, HDL-C, and triglyceride values after imputation. As LDL-C can only be calculated if triglycerides are <4.52 mmol/L (395.5 mg/dl), non-calculable LDL-C values were imputed. Kidney function is represented by eGFR calculated from creatinine, age, and sex.

### Statistical analysis

A logistic regression model was used to find risk factors discriminating between patients with proximal and patients with distal disease. A backward selection approach starting with the full model consisting of localization (proximal/distal), sex, age, BMI, diabetes mellitus, hypertension, smoking status, coronary artery disease, cerebrovascular disease, dyslipidemia, triglycerides, HDL-C, LDL-C, HbA_1c_, and calculated eGFR was performed based on the *p*-value from the likelihood method. A *p*-value of 0.2 was set as a criterion to keep variables during the model selection approach. The final model with laboratory values within 30 days of the intervention included group, sex, age, diabetes mellitus, hypertension, smoking status, dyslipidemia, LDL-C, triglycerides, HbA_1c_, and calculated eGFR.

Furthermore, three sensitivity analyses were performed using (i) a backward selection model with multiply imputed data with laboratory values within 180 days of the intervention, (ii) an available cases-based model (as used in the primary analysis), and (iii) a complete case data set based backward model selection approach.

Subgroup analyses were done for sex, smoking status, and diabetes mellitus. Therefore, the multiply imputed dataset was split into all categories of the respective subgroup (i.e., female and male sex; active, former, or never smoking; absence or presence of DM). Then, a backward selection as described above was performed in each of the individual data sets investigating which risk factors contribute to the distribution pattern within each subgroup separately. Thus, results should be interpreted for each subgroup separately. However, with this type of analysis, it cannot be concluded that an individual risk factor leads to a different distribution pattern stratified by the subgroup.

Patient characteristics of the study population are shown as mean ± standard deviation (SD) for continuous variables and as number with percentages for categorical variables. Results are shown as odds ratio (OR) with 95% confidence interval (CI). *p*-values <0.05 were accepted to indicate statistically significant differences between groups. All analyses were performed in R version 3.5.0 (2018 R Core Team, R Foundation for Statistical Computing, Vienna, Austria) with the package MICE for multiple imputation.

### Ethical approval

The study protocol conforms to the ethical guidelines of the 1975 declaration of Helsinki and has been approved by the institution's Ethics Committee on research involving human data waiving the need for individual patient consent prior to the year 2015 (approval 2018-00679). Written informed consent was obtained from each patient included in the study since the year 2015 when a general consent was introduced at our institution. This is a retrospective cross-sectional study originating from a consecutive registry of patients undergoing first-time endovascular treatment in a tertiary referral center (Bern University Hospital, Division of Vascular Medicine, Swiss Cardiovascular Center) from January 2000 until March 2018.

## Results

From a total of 637 patients (29% females) with first-time endovascular recanalization, angiographically relevant atherosclerosis was within the proximal (iliac) vascular territory in 351 (55%) and within the distal (infrageniculate) vascular territory in 286 (45%) patients, respectively (Table 1). Almost all cardiovascular risk factors were significantly different between these two patient groups. Patients with proximal PAD had a mean age of 64 years (males 63.1 years, females 66.5 years), while for distal PAD, the mean age was 74 years (males 73.2 years, females 76.3 years). Proximal disease was more prevalent with female sex and active smoking, whereas patients with distal disease were more frequent of higher age and had overweight, DM, arterial hypertension, and CAD (Table 1). With the exception of CRP (high number of missing values), all available laboratory parameters were included in the analyses and significantly differed between groups. In patients with proximal PAD, higher levels of total cholesterol, HDL-C, and LDL-C and triglycerides were determined, while higher levels of HbA_1c_ and creatinine (resulting in lower eGFR) were measured in the context of distal PAD (Table 1).

**Table 1:** Patient characteristics.

Parameter	Total (*n* = 637)		Proximal (*n* = 351)		Distal (*n* = 286)		Mean/risk difference	*p*-value
	*n*	Mean ± SD/*n* (%)	*n*	Mean ± SD/*n* (%)	*n*	Mean ± SD/*n* (%)	(95% CI)	
Age (years)	637	68.6 ± 12.5	351	64.3 ± 11.8	286	73.9 ± 11.2	−9.6 (−11.4 to −7.8)	<0.001
Female sex	637	185 (29.0%)	351	123 (35.0%)	286	62 (21.7%)	0.13 (0.07 to 0.20)	<0.001
Smoking status	631		347		284			<0.001
Active		254 (40.3%)		202 (58.2%)		52 (18.3%)	0.40 (0.33 to 0.47)	
Former		169 (26.8%)		93 (26.8%)		76 (26.8%)	0.00 (−0.07 to 0.07)	
Never		208 (33.0%)		52 (15.0%)		156 (54.9%)	−0.40 (−0.47 to −0.33)	
Arterial hypertension	631	514 (81.5%)	346	267 (77.2%)	285	247 (86.7%)	−0.10 (−0.15 to −0.04)	0.003
BMI (kg/m^2^)	595	26.4 ± 4.84	325	26.0 ± 4.56	270	27.0 ± 5.10	−1.00 (−1.78 to −0.22)	0.012
Dyslipidemia	620	424 (68.4%)	335	237 (70.7%)	285	187 (65.6%)	0.05 (−0.02 to 0.13)	0.19
Diabetes mellitus	633	247 (39.0%)	348	72 (20.7%)	285	175 (61.4%)	−0.41 (−0.48 to −0.34)	<0.001
Coronary artery disease	635	234 (36.9%)	349	110 (31.5%)	286	124 (43.4%)	−0.12 (−0.19 to −0.04)	0.002
Cerebrovascular disease	636	89 (14.0%)	350	41 (11.7%)	286	48 (16.8%)	−0.05 (−0.11 to 0.00)	0.08
Total-C (mmol/L)	507	4.65 ± 1.24	289	4.95 ± 1.25	218	4.26 ± 1.11	0.70 (0.49 to 0.91)	<0.001
LDL-C (mmol/L)	496	2.57 ± 1.04	281	2.77 ± 1.11	215	2.32 ± 0.88	0.45 (0.27 to 0.64)	<0.001
HDL-C (mmol/L)	532	1.25 ± 0.39	313	1.30 ± 0.40	219	1.18 ± 0.37	0.12 (0.05 to 0.19)	<0.001
Triglycerides (mmol/L)	508	1.91 ± 1.19	289	2.06 ± 1.34	219	1.72 ± 0.92	0.34 (0.14 to 0.55)	0.001
HbA_1c_ (%)	525	6.2 ± 1.4	294	5.9 ± 1.1	231	6.6 ± 1.6	−0.74 (−0.97 to −0.51)	<0.001
Creatinine (µmol/L)	635	108 ± 88	349	91 ± 65	286	129 ± 107	−38.2 (−51.7 to −24.7)	<0.001
eGFR (ml/min/1.73 m^2^)	635	71 ± 27	349	79 ± 24	286	61 ± 27	18.7 (14.7 to 22.7)	<0.001
C-reactive protein (mg/dl)	272	42.1 ± 52.9	95	25.2 ± 33.6	177	51.2 ± 58.8	−26.0 (−38.9 to −13.1)	<0.001

BMI, body mass index; eGFR, estimated glomerular filtration rate; HbA_1c_, glycated hemoglobin; HDL-C, high-density lipoprotein cholesterol; LDL-C, low-density lipoprotein cholesterol: Total-C, total cholesterol; CI, confidence interval.

Laboratory values were obtained within a time window of 30 days of the primary intervention.

### Main analysis

**Figure 1:**
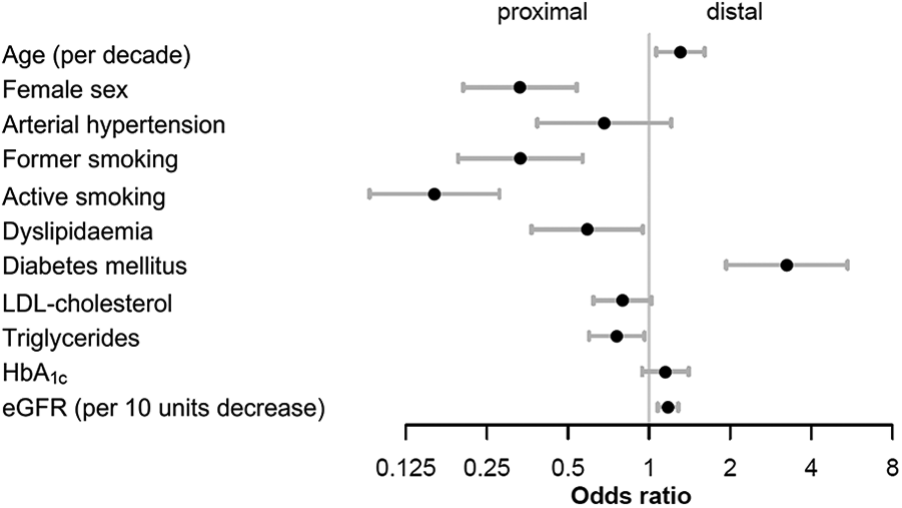
Main analysis for atherosclerosis distribution in the lower extremities. Odds ratios were calculated in the final model using backward selection on the multiply imputed data with laboratory values within 30 days of the intervention. eGFR, estimated glomerular filtration rate; HbA_1c_, glycated hemoglobin; LDL-cholesterol, low-density lipoprotein cholesterol.

Female sex, active or former smoker status, dyslipidemia, and higher levels of triglycerides were associated with proximal PAD (Figure 1, Supplementary Table). Older age, DM, and lower eGFR were associated with distal below the knee PAD (Figure 1, Supplementary Table). All other variables (arterial hypertension, body mass index, CAD, CVD, LDL-C, HDL-C, and HbA_1c_) were nonsignificant or dropped in the final model using backwards selection on the multiply imputed data.

### Subanalysis by sex

A total of 185 patients (29%) were female, and 452 patients were male (Table 1). While the distribution pattern was equal in males (228 with proximal, 224 with distal disease), the proximal distribution in females (*n* = 123) was more frequent than the distal distribution (*n* = 62). In female patients, proximal disease was strongly associated with active and former smoking, and arterial hypertension, whereas DM was associated with distal disease (Table 2A). In male patients, active and former smoking and hypertriglyceridemia were associated with proximal disease, and distal disease was associated with older age, DM, and lower eGFR (Table 2B).

**Table 2:** Odds ratios for atherosclerosis distribution in women (A) and men (B).

Parameter	Odds ratio (95% CI)	*p*-value
(A) Women		
Active smoking	0.03 (0.01 to 0.11)	<0.001
Former smoking	0.07 (0.02 to 0.26)	<0.001
Arterial hypertension	0.31 (0.10 to 0.96)	0.042
Dyslipidemia	0.46 (0.19 to 1.13)	0.09
Diabetes mellitus	3.45 (1.39 to 8.53)	0.008
LDL-C	0.64 (0.37 to 1.14)	0.12
(B) Men		
Age (per decade)	1.45 (1.14 to 1.85)	0.002
Active smoking	0.22 (0.12 to 0.41)	<0.001
Former smoking	0.50 (0.27 to 0.94)	0.03
Diabetes mellitus	3.29 (1.80 to 6.01)	<0.001
Cerebrovascular disease	0.61 (0.36 to 1.01)	0.06
LDL-C	0.83 (0.64 to 1.09)	0.18
Triglycerides	0.74 (0.58 to 0.96)	0.02
HbA_1c_	1.16 (0.94 to 1.44)	0.17
eGFR (per 10 units decrease)	1.19 (1.07 to 1.31)	<0.001

eGFR, estimated glomerular filtration rate; HbA_1c_, glycated hemoglobin; LDL-C, low-density lipoprotein cholesterol; CI, confidence interval.

Odds ratios were calculated for all parameters in the final model using backward selection on the multiply imputed data with laboratory values within 30 days of the intervention. An odds ratio <1 indicates a more proximal and an odds ratio >1 a more distal atherosclerosis distribution pattern.

### Subanalysis by smoking

Smoking status was reported as active in the majority of patients (40%) followed by never (33%) and former smokers (27%) (Table 1). While former smoking status was equally distributed, active smokers more often had proximal and never smokers distal PAD, respectively (Table 1). Female sex was strongly associated with proximal PAD both with active and former smoking, but not in never smokers (Table 3). Hypertriglyceridemia was associated with proximal atherosclerosis phenotype in active smokers, elevated LDL-C, or dyslipidemia with proximal disease in active and never smokers. DM was associated with distal disease in all smoking subgroups, older age in former and never smokers, and CKD in active smokers.

**Table 3:** Odds ratios for atherosclerosis distribution in active (A), former (B), and never (C) smokers.

Parameter	Odds ratio (95% CI)	*p*-value
(A) Active smoking		
Female sex	0.21 (0.08–0.56)	0.002
Arterial hypertension	0.50 (0.20–1.21)	0.12
BMI (per 5 units)	1.96 (1.29–2.99)	0.002
LDL-C	0.65 (0.43–0.99)	0.045
Triglycerides	0.57 (0.38–0.85)	0.007
HbA_1c_	1.49 (1.13–1.96)	0.004
eGFR (per 10 units decrease)	1.34 (1.15–1.56)	<0.001
(B) Former smoking		
Age (per decade)	1.51 (1.03–2.19)	0.033
Female sex	0.08 (0.02–0.27)	<0.001
Arterial hypertension	0.39 (0.12–1.30)	0.12
Dyslipidemia	0.23 (0.08–0.62)	0.004
Diabetes mellitus	5.76 (2.50–13.28)	<0.001
Triglycerides	0.71 (0.48–1.06)	0.09
eGFR (per 10 units decrease)	1.16 (0.99–1.36)	0.07
(C) Never smoking		
Age (per decade)	1.51 (1.04–2.20)	0.031
Female sex	0.55 (0.26–1.19)	0.13
Diabetes mellitus	4.85 (2.28–10.30)	<0.001
Cerebrovascular disease	0.50 (0.23–1.09)	0.08
LDL-C	0.65 (0.43–0.98)	0.042
eGFR (per 10 units decrease)	1.14 (0.97–1.34)	0.12

BMI, body mass index; eGFR, estimated glomerular filtration rate; HbA_1c_, glycated hemoglobin; LDL-C, low-density lipoprotein cholesterol; CI, confidence interval.

Odds ratios were calculated for all parameters in the final model using backward selection on the multiply imputed data with laboratory values within 30 days of the intervention. An odds ratio <1 indicates a more proximal and an odds ratio >1 a more distal atherosclerosis distribution pattern.

### Subanalysis by diabetes mellitus

The cohort included 247 patients (39%) with DM, 386 without DM, and 4 patients with unknown diabetes status (Table 1). Patients with unknown diabetes status were excluded from this subgroup analysis. Diabetes mellitus and atherosclerosis were not equally distributed with the proximal atherosclerosis distribution pattern (276 proximal vs. 110 distal) being more frequent in the absence of DM and the distal pattern (175 distal vs. 72 proximal) being more frequent in diabetic patients. In the group without DM, nearly all risk factors identified in the main analysis were associated with a proximal distribution pattern. Older age and lower eGFR were associated with a distal distribution pattern of PAD (Table 4A). Thereby, female sex and active or former smoking again showed the strongest association with the proximal distribution pattern of PAD (Table 4A). Similarly, in the group with DM, female sex, active or former smoking, and elevated triglycerides were associated with a proximal, whereas older age and lower eGFR correlated with a distal distribution pattern of PAD (Table 4B). Overall, female sex and active or former smoking show the strongest association with proximal distribution of PAD.

**Table 4:** Odds ratios for atherosclerosis distribution in the absence (A) and presence (B) of diabetes mellitus.

Parameter	Odds ratio (95% CI)	*p*-value
(A) Absence of diabetes mellitus		
Age (per decade)	1.36 (1.06–1.75)	0.016
Female sex	0.38 (0.21–0.69)	0.001
Active smoking	0.19 (0.09–0.38)	<0.001
Former smoking	0.37 (0.19–0.72)	0.004
Arterial hypertension	0.55 (0.29–1.05)	0.07
Dyslipidemia	0.51 (0.29–0.91)	0.02
LDL-C	0.75 (0.56–1.01)	0.06
eGFR (per 10 units decrease)	1.12 (1.00–1.25)	0.05
(B) Presence of diabetes mellitus		
Age (per decade)	1.55 (1.04–2.30)	0.03
Female sex	0.26 (0.11–0.65)	0.004
Active smoking	0.12 (0.05–0.31)	<0.001
Never smoking	0.34 (0.14–0.84)	0.02
BMI (per 5 units)	1.45 (0.98–2.13)	0.06
Cerebrovascular disease	0.54 (0.25–1.14)	0.10
Triglycerides	0.58 (0.38–0.87)	0.008
HbA_1c_	1.28 (0.99–1.66)	0.06
eGFR (per 10 units decrease)	1.27 (1.09–1.49)	0.003

BMI, body mass index; eGFR, estimated glomerular filtration rate; HbA_1c_, glycated hemoglobin; LDL-C, low-density lipoprotein cholesterol; CI, confidence interval.

Odds ratios were calculated for all parameters in the final model using backward selection on the multiply imputed data with laboratory values within 30 days of the intervention. An odds ratio <1 indicates a more proximal and an odds ratio >1 a more distal atherosclerosis distribution pattern.

Sensitivity analyses as described in the Materials and methods section reproduced consistently the findings of the primary analysis for female sex, active or former smoking, dyslipidemia, and higher triglyceride levels to be associated with proximal PAD. Similarly, DM and CKD were associated with distal PAD. The other factors were not or not conclusively associated with atherosclerosis segregation.

## Discussion

The present cross-sectional study of consecutive symptomatic PAD patients shows a distinct cardiovascular risk factor profile in relation to the peripheral vascular atherosclerosis pattern. The main finding is that female sex, particularly in the context of active or former smoking, has the strongest association with proximal PAD (Supplementary Figure). On the other hand, distal PAD is significantly associated with older age, diabetes mellitus, and chronic kidney disease (Supplementary Figure). Even within subanalyses, statistical significance remained suggesting a differential site-specific effect of some of the risk factors that need further investigation. Except for elevated triglycerides that seem to promote proximal atherosclerosis, other lipoproteins, BMI, and hypertension had no effect on proximal or distal atherosclerosis predilection sites in PAD.

The finding that women are more susceptible for proximal atherosclerosis segregation in iliac arteries is intriguing because to date there is a paucity of data on sex differences in PAD regarding risk profiles, atherosclerotic phenotypes, management, and outcomes for women (18). Furthermore, our findings indicate that males and females may have different pathomechanisms and patterns of atherosclerotic development (19). One explanation is that in these studies, peripheral vascular disease has been considered one single disease entity and thus the observed sex-specific effects on atherosclerosis segregation may have been concealed. The underlying pathomechanisms for the observed sex differences remain unclear.

Our study results are in line with previous observations and studies that demonstrated an association of female sex (20–22), smoking (20, 21, 23–26), and dyslipidemia (20) with a proximal atherosclerosis phenotype, and older age (13, 20, 23, 24), male sex (20, 23, 27), diabetes mellitus (13, 20, 23–26), and chronic kidney disease (14, 24) with a distal atherosclerosis phenotype. Furthermore, we have confirmed that some of these factors, e.g., chronic kidney disease and diabetes, are already segmentally effective in a targeted population with first-time manifestation of clinically relevant symptoms.

In contrast to the above studies, we have chosen a unique targeted approach with (i) isolated anatomic phenotypes, i.e., proximal or distal atherosclerosis distribution pattern, and with (ii) clinical relevance of disease expression, i.e., indication for endovascular recanalization. The targeted approach was preferred in order (i) to reduce confounding and elucidate more clearly cardiovascular risk factors that may be associated with the segregation into a proximal or a distal atherosclerosis expression, (ii) to detect factors with even small effect size, and (iii) to provide unequivocal phenotype classification, which was not consistent in some of the previous studies.

Our targeted study design is supplementary to the all-comer design of two previous studies on atherosclerosis localization and might explain at least in part that in these, female sex was only associated with femoropopliteal but not aortoiliac atherosclerosis expression (23, 27). Furthermore, one study focused on patients with critical limb ischemia (CLI) only (27).

Neither did we find an association with a proximal disease type in patients with CAD, CVD, or hypertension as shown in another PAD all-comer study (20). The major limitation of that and of further studies (22, 25) was that predilection sites were only controlled by changes of continuous-wave Doppler spectrum and not by directly imaging atherosclerosis burden and distribution as in the present study, i.e., angiography and duplex sonography prior to the intervention.

The inconsistent classification of proximal and distal disease location and consideration of multilevel disease are further limitations of some studies that might account for the observed differences on atherosclerosis manifestation (13, 14, 20–27).

Although atherosclerotic cardiovascular disease entities, i.e., PAD, CAD, and CVD, share the same modifiable cardiovascular risk factors, e.g., arterial hypertension, DM, hyperlipidemia, or smoking (12, 28, 29), which impinge on the entire vascular bed, different phenotypes of atherosclerosis expression are observed among different vascular territories but also solely within the peripheral vascular bed itself (5–11, 30). This is probably not explained by local site-specific anatomical, histological, and hemodynamic differences only. The rational question, therefore, arises whether there are further relevant genetic, epigenetic, and mechanistic pathways that differ between vascular beds. Atherosclerosis is a chronic inflammatory process of the arterial wall initiated by endothelial dysfunction and deposition of lipoproteins (1). However, numerous pathophysiological stimuli are involved in endothelial dysfunction and activation, the initial step of atherogenesis, e.g., reduced nitric oxide bioavailability, oxidative stress, proinflammatory cytokines, infectious agents, disturbed glucose metabolism, and hemodynamic forces (31, 32). These originate from various pathways, e.g., hemostasis, inflammation, and diabetes mellitus. Interestingly, the vascular endothelium displays an antigenic heterogeneity with vascular bed-specific expression of receptors involved in the above pathways, which might contribute to the phenotypic atherosclerosis segregation (33–36). This is in line with recent results from a genome-wide association study (GWAS) (Million Veteran Program) that indicate an important role of genes related to smoking, i.e., nicotine dependence [cholinergic receptor nicotinic alpha 3 subunit (CHRNA3)] and to thrombosis [factor V (F5 p.R506Q)] in the specific segregation of atherosclerosis in peripheral arteries and development of PAD (37).

Despite the described differences in atherosclerosis expression, therapeutic options are the same among PAD, CAD, and CVD patients in current clinical practice guidelines (12, 38, 39). Moreover, PAD is considered a single disease entity not taking into account specific segregation patterns so far (12, 38). This may in fact explain why several large randomized controlled trials in patients with PAD have had limited success in reducing major adverse cardiovascular event (MACE) further and numbers of MACE still remain substantial (40–42). This is when therapies are not specifically tailored and will miss their optimal effect. On the contrary, modulating PAD-associated pathways more specifically, i.e., factor Xa-inhibition (as mentioned above, the role of coagulation factors in the establishment of PAD has been demonstrated with GWAS) (37) has a great impact on reducing MACE in PAD patients as demonstrated by positive results of two recent large clinical outcome studies (COMPASS and VOYAGER PAD) (43, 44). Consideration of different atherosclerosis phenotypes in PAD patients might also be of interest because different phenotypes are associated with different mortality rates (45).

Our findings may have important clinical implications. We have identified at least two anatomic phenotypes within the population of PAD patients that show a proximal and a distal atherosclerosis distribution pattern, respectively. We suggest a sex-specific and possibly genetically determined susceptibility to different atherogenic risk factors even in different segments of the peripheral arteries. Thus, clarifying the underlying mechanisms and pathways related to specific PAD phenotypes may allow sex-specific screening programs and primary prevention and therapy modifications in the future. However, further studies are needed to (i) assess these two different types of PAD are clinically meaningful and (ii) establish whether these associated risk factors are causal and can thus be intervened upon to prevent local PAD/atherosclerosis development.

Strengths of the present study are the large number of patients with isolated anatomic phenotypes of proximal or distal atherosclerosis expression, the comprehensive amount of clinical data, and the instant direct angiographic phenotype confirmation. However, several limitations have to be considered. The atherosclerotic phenotype was determined according to clinically driven indications for revascularization. This does not take into account the beginning and temporal aspects of atherogenic changes as well as information on early atherosclerotic lesions limiting generalizability. Considering only isolated anatomic phenotypes in a multifactorial disease may represent an oversimplification. Although the lack of temporality resulting from the cross-sectional design and outcomes data is a limitation, the study design represents one possible approach to reduce confounding and increase sensitivity for possible signals. Completeness of medical records and laboratory results is a limitation of any retrospective study. In this study, information on estrogen replacement therapy and values of C-reactive protein (no pre-interventional routine parameter) is largely missing. Although dyslipidemia was associated with proximal atherosclerosis expression, it considered any type of dyslipidemia and did not distinguish between treated and untreated patients and did not take into account treatment effect sizes and was, therefore, not an overly discriminating parameter. Because our patients were almost exclusively White individuals, the effects of different races and ethnicities cannot be assessed in our study.

In conclusion, the present study identified female sex, particularly in the context of cigarette smoking, as the main risk factor associated with proximal atherosclerosis expression in the lower limbs. Higher age, DM, and CKD are associated with distal atherosclerosis expression. Our data suggest a sex-specific and individual susceptibility to atherogenic risk factors, and that PAD has at least two different anatomic phenotypes. This may have a potential clinical impact on primary and secondary prevention measures.

## Data Availability

The raw data supporting the conclusions of this article will be made available by the authors, without undue reservation.
