# Supplementary material for: Association of sex and cardiovascular risk factors with atherosclerosis distribution pattern in lower extremity peripheral artery disease
Source: Front Cardiovasc Med. 2023 Jun 27;10:1004003. doi: 10.3389/fcvm.2023.1004003 (PMC10333498; doi:10.3389/fcvm.2023.1004003)
Supplement: Supplementary file 1 [file Table1.docx]

**Supplemental Table. Odds ratios for atherosclerosis distribution for all parameters**

| Parameter | Odds ratio (95% CI) | *P*-value |
| --- | --- | --- |
| Age (per decade) | 1.31 (1.06 to 1.61) | .011 |
| Female sex | 0.33 (0.20 to 0.54) | < .001 |
|  |  |  |
| Active smoking | 0.16 (0.09 to 0.28) | < .001 |
| Former smoking | 0.33 (0.20 to 0.57) | < .001 |
|  |  |  |
| Arterial hypertension | 0.68 (0.38 to 1.21) | .19 |
| Dyslipidemia | 0.59 (0.37 to 0.95) | .029 |
| Diabetes mellitus | 3.25 (1.93 to 5.46) | < .001 |
| LDL-C | 0.80 (0.62 to 1.02) | .07 |
| Triglycerides | 0.76 (0.60 to 0.96) | .021 |
| HbA_1c_ | 1.15 (0.94 to 1.40) | .16 |
| eGFR (per 10 units decrease) | 1.18 (1.08 to 1.28) | < .001 |
| Odds ratios were calculated in the final model using backwards selection on the multiply imputed data with laboratory values within 30 days of the intervention. An odds ratio <1 indicates a more proximal and an odds ratio >1 a more distal atherosclerosis distribution pattern.  eGFR = estimated glomerular filtration rate; HbA_1c_ = glycated hemoglobin; LDL-C = low-density lipoprotein cholesterol | | |
