# Supplementary material for: Association of sex and cardiovascular risk factors with atherosclerosis distribution pattern in lower extremity peripheral artery disease
Source: Front Cardiovasc Med. 2023 Jun 27;10:1004003. doi: 10.3389/fcvm.2023.1004003 (PMC10333498; doi:10.3389/fcvm.2023.1004003)
Supplement: Supplementary file 2 [file Table2.docx]

**Risk factors and their influence on segmental atherosclerosis expression**
